# Characteristics of Hepatocellular Carcinoma by Sex in Mexico: A Multi-Institutional Collaboration

**DOI:** 10.3390/diseases12100262

**Published:** 2024-10-21

**Authors:** Javier Melchor-Ruan, Luis Santiago-Ruiz, Blanca Olivia Murillo-Ortiz, Samuel Rivera-Rivera, Yelda A. Leal-Herrera, David Suárez-García, José María Remes-Troche, Peter Grube, Gustavo Martínez-Mier, Erika Ruiz-García, Alan Ramos-Mayo, José Antonio Velarde-Ruiz-Velasco, Ricardo Gamboa-Gutierrez, Karla Gabriela Ordoñez-Escalante, Laura Esthela Cisneros-Garza, Pilar Leal-Leyte, Jesús Sepúlveda-Delgado, María Saraí González-Huezo, Ricardo Arvizu-Castillo, Jorge Urías-Rocha, Celia Beatriz Flores-de-la-Torre, Leonardo Manuel Carrillo-Mendoza, Juan Manuel Gámez-del-Castillo, Martín Lajous, Adriana Monge, Daniel Zamora-Valdés

**Affiliations:** 1 Departamento de Gastroenterología, Instituto Nacional de Cancerología, Ciudad de México 14080, Mexico; jmelchorr@incan.edu.mx (J.M.-R.); eruizg@incan.edu.mx (E.R.-G.); ramosmayo.md@tec.mx (A.R.-M.); 2 Centro de Investigación en Salud Poblacional, Instituto Nacional de Salud Pública, Cuernavaca 62100, Mexico; cisp48@insp.mx (L.S.-R.); mlajous@insp.mx (M.L.); 3 Unidad de Investigación en Epidemiologia Clínica, UMAE No. 1, OOAD, Instituto Mexicano del Seguro Social, Guanajuato 37328, Mexico; blanca.murillo@imss.gob.mx; 4 División de Atención Oncológica en Adultos, Coordinación de Atención Oncológica, Instituto Mexicano del Seguro Social, Ciudad de México 03020, Mexico; samuel.rivera@imss.gob.mx; 5 Registro Poblacional de Cáncer Mérida, Unidad de Medicina de Alta Especialidad UMAE Mérida, Instituto Mexicano del Seguro Social, Mérida 97155, Mexico; yelda.leal@imss.gob.mx; 6 Centro Institucional de Capacitación y Registro de Cáncer, Coordinación de Investigación en Salud, CMN SXXI del IMSS, Ciudad de México 06720, Mexico; 7 Unidad de Medicina de Alta Especialidad UMAE No. 1, Instituto Mexicano del Seguro Social, León de Los Aldama 37328, Mexico; david.suarez@imss.gob.mx; 8 Universidad Veracruzana, Veracruz 94294, Mexico; joremes@uv.mx (J.M.R.-T.); pgrube@uv.mx (P.G.); gustavo.martinezmi@imss.gob.mx (G.M.-M.); 9 Unidad de Medicina de Alta Especialidad UMAE Hospital de Especialidades 14, Instituto Mexicano del Seguro Social, Veracruz 91810, Mexico; 10 Hospital Civil Guadalajara Fray Antonio Alcalde, Guadalajara 44280, Mexico; antonio.velarde@academicos.udg.mx; 11 División de Oncología, Unidad de Medicina de Alta Especialidad UMAE Mérida, Instituto Mexicano del Seguro Social, Mérida 97155, Mexico; ricardo.gamboa@imss.gob.mx; 12 Laboratorio de Anatomía Patológica, Unidad de Medicina de Alta Especialidad UMAE Mérida, Instituto Mexicano del Seguro Social, Mérida 97155, Mexico; karla.ordonez@imss.gob.mx; 13 Christus Muguerza Alta Especialidad, Monterrey 64060, Mexico; laura_cisneros@yahoo.com; 14 Centro Médico Naval, CEMENAV, Ciudad de México 04470, Mexico; cemenav.ensenanza@naval.sm; 15 Hospital Regional de Alta Especialidad Ciudad Salud, Tapachula 30830, Mexico; jesussepulveda@redmexei.mx; 16 Centro Médico Toluca—ISSEMyM, Toluca de Lerdo 52170, Mexico; msarai.gonzalez@issemym.gob.mx; 17 Unidad de Quemados, Hospital General de Zona # 14, Instituto Mexicano del Seguro Social, Hermosillo 83120, Mexico; ricardo.arvizu@imss.gob.mx (R.A.-C.); carlos.avilesg@imss.gob.mx (J.U.-R.); 18 Centro Estatal de Oncología de Campeche, Campeche 24096, Mexico; cflores@oncologiacampeche.gob.mx (C.B.F.-d.-l.-T.); leonardocarrillo@oncologiacampeche.gob.mx (L.M.C.-M.); 19 Hospital General del Estado de Sonora “Dr. Ernesto Ramos Bours”, Hermosillo 83000, Mexico; j.gamez@isssteson.gob.mx; 20 Hepatobiliary Sciences and Liver Transplantation, KASCH, KAMC, MNGHA, Ar Rimayah, Riyadh 14611, Saudi Arabia; valdesda1@mngha.med.sa

**Keywords:** hepatocellular carcinoma, cancer epidemiology, sex differences, Mexico

## Abstract

Liver cancer is the fourth leading cause of cancer-related death worldwide. In Mexico, there is a high burden of liver cancer mortality in rural states, affecting both women and men equally. Thus, we aimed to describe the demographic and clinical characteristics of hepatocellular cancer (HCC) by sex in Mexico. Demographic and clinical information was extracted retrospectively from the medical records of patients with HCC initially treated (2015–2022) at institutions participating in a national survey across the country. The male-to-female ratio was calculated at the national and regional levels, and the results were stratified by sex. Among 697 HCC patients, the age at diagnosis was 65.4 ± 11.9 years and 20% were diagnosed at ≥75 years. The male-to-female ratio was 1.4:1, ranging from 1:1 in the northwestern and southwestern regions, to 2.1:1 in the western region. The proportion of cirrhosis was similar between the sexes; however, the etiology of cirrhosis differed: cryptogenic cirrhosis was higher in women and alcohol consumption was higher in men. Men had a higher proportion of advanced HCC, poor/undifferentiated tumors, and ≥4 nodules than women. HCC in the Mexican population affects both men and women at a 1.4:1 male-to-female ratio. This unique proportion by sex could be explained by the differences in the prevalence of risk factors across our heterogeneous country.

## 1. Introduction

Liver cancer is the fourth leading cause of cancer-related death worldwide [1]. The risk factors for hepatocellular carcinoma (HCC), the most common type of liver cancer, include hepatitis B virus (HBV) and hepatitis C virus (HCV) infections, excessive alcohol consumption, aflatoxin B1 (AFB1) exposure, and metabolic dysfunction-associated fatty liver disease (MAFLD) [2]. In the pathogenesis of HCC, most risk factors lead to cirrhosis, and this damage can be accelerated in genetically susceptible individuals [2]. 

In Mexico, the HBV and HCV seroprevalence is low, while the proportion of the population reporting excessive alcohol consumption is moderate (18%) and higher in men than in women (30% vs. 6·2%) [3,4,5]. Alcoholic liver disease was the second most common etiology of cirrhosis in a convenience sample from tertiary care hospitals in Mexico [6]. Aflatoxin B1 (AFB1) is an established liver carcinogen and a frequent product of fungal contamination in maize, a staple food of Mexican and Central American populations [7]. Recently, in Mexico, AFB1 exposure was shown to be highly prevalent (>90% detection) at moderate levels of exposure [8]. Finally, in Latin America, there has been an increase in the number of HCC cases related to MAFLD [9].

HCC in Mexico has a unique epidemiological pattern because HCC mortality affects women and men at a 1:1 male-to-female ratio, as opposed to the global 2.7:1 ratio [10,11,12]. Also, a higher disease burden has been observed in rural areas [10]. There is limited information regarding the distribution of risk factors and the clinical characteristics of women and men in Mexico. Thus, this study explored the differences in the risk factors and clinical characteristics of men and women diagnosed with HCC in Mexico.

## 2. Materials and Methods

### 2.1. Mexican Interdisciplinary Network for Hepatocellular Cancer Research (RIMICH)

In November 2021, a multidisciplinary and multi-institutional consortium to study hepatocellular cancer (RIMICH, Red Interdisciplinaria Mexicana para la Investigación en Cáncer Hepatocelular) was established, and its main objective was to identify the regions in Mexico with HCC cases to analyze aflatoxin exposure. This collaboration convened various specialists, including gastroenterologists, hepatologists, radiologists, epidemiologists, public health personnel, surgical oncologists, transplant surgeons, hepatopancreatobiliary surgeons, and pathologists, across the country. As Mexico does not have a national cancer registry, individual efforts regarding HCC are warranted. The data from this study are a RIMICH resource and are available to other researchers upon request.

The Research, Ethics, and Biosecurity Committees at the National Institute of Public Health (INSP), National Cancer Institute (INCan), Naval Medical Center (CEMENAV), Mexican Institute of Social Security (IMSS), and the Institute of Social Security of the State of Mexico and its Municipalities (ISSEMYM) evaluated and approved the study protocol (INSP Study Protocol 1700).

### 2.2. Medical Record Review and Data Extraction

In November 2021, an invitation was sent to members of RIMICH and members of the Mexican Hepato-Pancreato-Biliary Association, Mexican Hepatology Association, and Mexican Gastroenterology Association. Physicians from 12 different centers responded to the survey. Demographic and clinical information from medical records was extracted from patients diagnosed with HCC initially treated at participating institutions between 2015 and 2022. Not all participating institutions had electronic medical records; thus, manual clinical data abstraction was conducted over a 16-month period. The data abstraction form was based on the forms used for cancer registries in the country [13], which are based on the International Agency for Research on Cancer guidelines for Cancer Registries in low- and middle-income countries [14]. Other variables were added, such as cirrhosis diagnosis and etiology, as well as diagnostic methods. The final version was reviewed by the RIMICH participants.

The final data abstraction form was then shared with all participating institutions, where medically trained personnel filled out the form. The study data were collected and managed using REDCap electronic data capture tools hosted at the Asociación Mexicana Hepatopancreatobiliar [15]. Data were centrally reviewed (JMR, LSR, and AM), and inconsistencies and implausible values were resolved by treating facility personnel. The participants’ age at diagnosis in years was categorized (˂50, 50–59, 60–69, ≥70 years). The participants were categorized according to their sex at birth as female or male. Participants were categorized according to eight regions in Mexico: Northwest (Sonora, Sinaloa, and Durango), northeast (Coahuila, Nuevo León, and Tamaulipas), west (Michoacán and Jalisco), east (Veracruz, Puebla, Tlaxcala, and Hidalgo), center-north (Zacatecas, San Luis Potosí, Querétaro, and Guanajuato), central-south (Mexico City, State of Mexico, and Morelos), southwest (Guerrero, Oaxaca, and Chiapas), and southeast (Yucatán, Campeche, Quintana Roo, and Tabasco). The patients’ height and weight were measured at diagnosis. The body mass index (BMI) was calculated as weight in kilograms divided by height in meters squared (kg/m^2^), and patients were categorized as overweight/obese when their BMI was ≥25 kg/m^2^. Participants with a clinical diagnosis of cirrhosis were identified along with the etiology presumed by the treating physician [alcohol, HBV virus, HCV virus, MAFLD, autoimmune hepatitis, other etiologies, or unknown]. Patients without a known cause of cirrhosis were defined as having cryptogenic cirrhosis [16]. The liver function Child–Pugh score and clinical stage were also extracted using the Barcelona Clinic Liver Cancer (BCLC) staging system [17,18,19]. Patients were categorized according to alpha-fetoprotein (AFP) levels of <20, 20–399, and ≥400 ng/mL, representing normal, moderately elevated, and markedly elevated AFP levels, respectively [20]. The tumor types [(a) classic types: trabecular, trabecular acinar, acinar, pseudoglandular; (b) fibrolamellar; (c) clear cell; (d) other; and (e) undetermined] and tumor differentiation (well, moderate, poor to undifferentiated, unspecified) were obtained from histopathology reports. Data on imaging reports included information on the number of nodules (1, 2–3, ≥4, unspecified), the size of the largest nodule, the presence of a nodule > 5 cm, metastases, and/or extrahepatic disease. Information on the different treatment modalities was obtained and categorized as follows: (a) curative/response-intent: ablation, surgery, TAE/TACE, other/combined, (b) palliative or systemic therapy, and (c) no treatment.

### 2.3. Statistical Analyses

Continuous and categorical variables are summarized as mean ± standard deviation (SD) or median (quartile 1–quartile 4 range) and percentages. The male-to-female ratio was calculated at national and regional levels. In addition, all results were stratified according to sex. The percentage of missing information for all analyzed characteristics has been reported. Statistical differences were not tested for, but rather explored to determine whether the magnitude of the observed difference was clinically meaningful [21]. Data management and analyses were performed using the Statistical Analysis Systems software package (version 9.4; SAS Institute Inc., Cary, NC, USA).

## 3. Results

**Figure 1:**
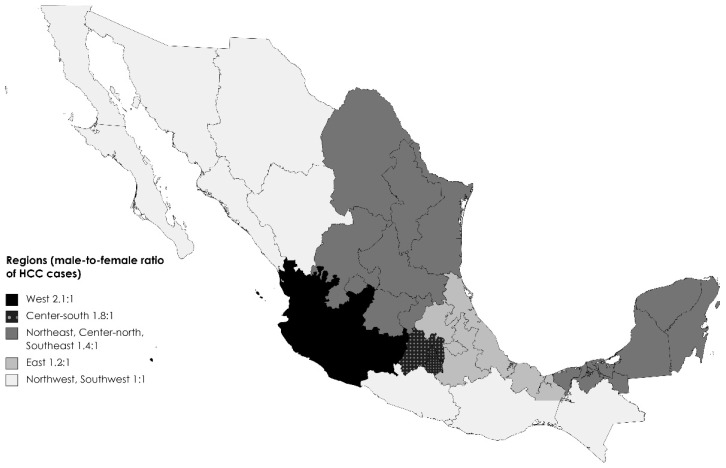
Male-to-female ratio of hepatocellular carcinoma (HCC) by region in Mexico. West (*n* = 56), center-south (*n* = 138), northeast (*n* = 24), center-north (*n* = 48), southeast (*n* = 1.4:1), east (*n* = 174), northwest (*n* = 32), southwest (*n* = 76). States with no participants: Baja California, Baja California Sur, Chihuahua, Nayarit, Aguascalientes, and Colima.

Data were obtained for 784 patients with HCC, and 87 patients were excluded because their diagnosis was made prior to 2015. Of the remaining 697 patients, the mean (±SD) age at diagnosis was 65.4 (±11.9) and 20% were diagnosed at ≥75 years of age (Mexico’s life expectancy) [22]. Male participants represented 57.8% (*n* = 403), and the male-to-female ratio was 1.4:1. However, this ratio differed across regions, from 1:1 in the northwest and southwest to 2.1:1 in the west (Figure 1).

The patients’ age at diagnosis was slightly higher in women than in men (>60 years: 78% women vs. 73% men; Table 1). While the proportion of cirrhosis was similar across sexes (overall 67%), the proportion of women with an unknown etiology of cirrhosis was much higher than that of men (51.1% vs. 20.7%). In addition, the HCV and MAFLD levels were much higher in women than in men, while almost 60% of cirrhosis cases in men were attributed to alcohol consumption. However, information on cirrhosis etiology was missing for 35% of the patients. Men had a higher proportion of advanced HCC (BCLC: C-D) than women. Liver dysfunction (Child-Pugh B/C), the AFP levels at diagnosis, and the tumor types were similar between men and women. Approximately 60% of patients were biopsied to complete the diagnostic process, and more than 70% received some type of treatment.

**Figure 2:**
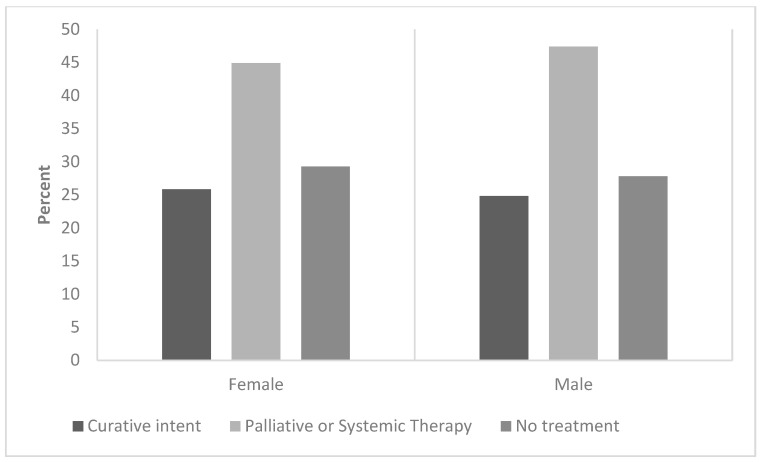
The figure shows the percentage of female and male patients with hepatocellular carcinoma by the type of treatment they received: curative intent, palliative or systemic therapy, and no treatment.

Men had a higher proportion of poor/undifferentiated HCC histologic-grade tumors (women: 2.2% vs. men: 16.5%) (Table 2). Interestingly, the incidence of multinodular disease (≥4 nodules) was twice as high in men than in women (14.9% vs. 7.5%, respectively). However, the frequency of metastases and/or extrahepatic disease at diagnosis was similar in women and men. The type of treatment was similar across the sexes (Figure 2). We found that curative/response-intended care was sought by 26% of female patients with HCC and 25% of male patients with HCC. Approximately 50% of patients with HCC (both women and men) received systemic therapy or palliative care alone.

## 4. Discussion

In this large series study of hepatocellular carcinoma in Mexico, the male-to-female overall ratio was 1.4:1, with several important clinical differences across the sexes. These numerous clinical differences might explain the unique epidemiological pattern of hepatocellular carcinoma in Mexico (male-to-female ratio, 1.4:1). Globally, HCC affects men more than women [23]. In contrast to the 2.7:1 world-average male-to-female ratio, in our study, the ratio ranged from 1:1 to 2.1:1, which is consistent with previous reports on liver cancer mortality in Mexico [10,11,12]. This is significantly different from the ratio in high-income countries (2.8:1, on average) [23].

The reason HCC affects men and women across the country in such a different ratio might be explained by the differences in the prevalence of risk factors in women compared to men.

In 2018, a small retrospective study (*n* = 148) was conducted using a convenience sample from two states in Mexico [24]. Similar to our findings, cryptogenic cirrhosis was the most common etiology of cirrhosis, followed by alcohol consumption and HVC. In both studies, more than half of the patients had advanced HCC (BCLC: C-D) at diagnosis, most tumors were larger than 5 cm, and more than 70% of the patients had AFP levels >20 ng/mL. The differences between the studies are likely due to regional and sample size limitations. Furthermore, since Cisneros Garza et al. did not stratify their results by sex, the studies are not comparable.

A large HCC series in Brazil found that, among approximately 1400 patients, liver cirrhosis was the most common risk factor for HCC (98% cirrhosis), mainly due to HCV infection [25]. Similarly, another South American study found that liver cirrhosis, mainly HCV, was the most common risk factor (85% of cirrhosis cases) [26]. In our study, cirrhosis was an important risk factor for HCC (67%) in both women and men, mainly due to excessive alcohol consumption in men and cryptogenic cirrhosis in women. HBV and HCV seroprevalence is low in Mexico (HB surface antigens: 0.51%; anti-HCV antibodies: 0.38%), and the reported incidence is lower (0.28 and 1.06 per 100,000, respectively) [27] than the global average [28]. Our results showed that viral-induced cirrhosis is a more common risk factor for HCC in women than in men in Mexico.

Alcohol cirrhosis was five times more prevalent in men than in women, which is consistent with the alcohol consumption patterns published elsewhere [29]. While excessive alcohol consumption in Mexico is a public health problem, its prevalence is lower than that in high-income countries such as the USA (18 vs. 29%, respectively), and differences between men and women are more pronounced (Mexico men: 30% vs. women: 6.2%; USA men: 44.7% vs. women: 13.1%) [29].

In our study, MAFLD cirrhosis was twice as prevalent in women compared to men. This is consistent with the higher prevalence of overweight/obesity among women in both our study and the national data (ENSANUT overweight/obesity: 75.0% women, 69.6% men) [30]. Other countries have shown sex differences in MAFLD prevalence, but in contrast to our findings, in these countries, men had the highest prevalence [31]. Further, in our study, 62.1% of women with cryptogenic cirrhosis were overweight/obese, and evidence suggests that cryptogenic cirrhosis in the presence of metabolic abnormalities is most likely due to MAFLD [16].

The tumor size was similar across the sexes. According to histological reports, most women and men have one nodule [25]. Other important characteristics differed between the women and men. Women were less likely to be diagnosed at advanced BCLC stages, have poorly undifferentiated tumors, and have ≥4 nodules [32]. The most common therapies for HCC were similar across sexes; most patients received palliative care/systemic therapy or no treatment. These data suggest that the diagnosis of HCC still frequently occurs at advanced stages of the disease in Mexico, where curative-intent therapy is no longer an option, particularly in men.

Over the years, there have been multiple efforts to create a national cancer registry in the country; however, they have not yet come to fruition. Thus, researchers and clinicians have attempted to create this network for liver cancer research, which began during the SARS-CoV-2 pandemic, to generate scientific evidence on the relevance of different hepatocellular cancer risk factors (such as aflatoxins) in our population.

Our study has several strengths. To our knowledge, this is the first study focusing on sex differences. It is also the largest study to date in Mexico and one of the largest in Latin America. It also includes the participation of 12 different institutions from different health providers in Mexico covering a 7-year period. However, this is a convenience sample in which only patients who seek and receive treatment have medical records. Because this was a retrospective study, we were limited to the information available in the medical records. For example, in some hospitals in the country, when patients were referred to cancer referral centers, imaging studies were not repeated because of other clinical characteristics of the disease, and we had missing information on several sections of the questionnaire, potentially affecting the tumor staging accuracy. Since this study was based on medical records, information on access to healthcare and the willingness to search for healthcare was not available, nor was information regarding socioeconomic and lifestyle characteristics that could explain regional differences. Although this is the largest HCC series in Mexico, regional differences could be explained by the smaller sample size after stratification. However, on average, the male-to-female ratio in this study was 1.4:1, compared to the global ratio of 2.7:1. Additionally, not all cancer centers participated in the study; therefore, this information may not be applicable to all centers in the country, even though major cancer referral centers participated and most states in the country were represented (Figure 1).

## 5. Conclusions

HCC in this population affects both men and women at a 1.4:1 male to female ratio. The lower male-to-female ratio in Mexico could be explained by sex differences in the prevalence of HCC risk factors. Men present with more advanced disease and a worse prognosis. Further research is needed to explore other factors contributing to the observed sex ratios and develop early detection strategies for HCC in the Mexican population.

## Figures and Tables

**Table 1:** Clinical and demographic characteristics of men and women with hepatocellular carcinoma in Mexico, from 2015 to 2022 *.

	Female		Male	
	(*n* = 294)	Missing, %	(*n* = 403)	Missing, %
Mean age at diagnosis, years	66.3(12.1)	0	64.8(11.7)	0
<50	7.1		10.7	
50–59, %	14.3		15.9	
60–69, %	34.0		37.2	
70+, %	44.6		36.2	
Mean body mass index, kg/m^2^	26.9(5.2)	0	26.1(4.3)	0
Overweight/Obese, %	61.2	0	59.1	0
Cirrhosis, %	65.5	0	68.5	0
Cirrhosis etiology		36.7		33.0
Unknown, %	51.1		20.7	
Alcohol, %	10.8		59.6	
Hepatitis C virus, %	20.4		12.6	
MAFLD, %	8.6		3.0	
Hepatitis B virus, %	3.8		3.3	
Autoimmune Hepatitis, %	2.7		0.7	
Other, %	2.7		0.0	
Child Score		35.4		32.8
A, %	58.4		54.2	
B, %	31.6		32.1	
C, %	7.9		11.1	
Biopsy, %	59.4	0	56.2	0
Treated, %	70.4	0	72.2	0

* Values are mean (SD) for continuous variables and percentage (95% confidence interval) for categorical variables. The values of the categorical variables may not add up to 100% because of rounding. Abbreviations: metabolic dysfunction-associated fatty liver disease (MAFLD).

**Table 2:** Tumor characteristics of men and women with hepatocellular carcinoma in Mexico, from 2015 to 2022 *.

	Female (*n* = 294)		Male (*n* = 403)	
		Missing, %		Missing, %
Differentiation		41.5		44.4
Well, %	25.6		30.8	
Moderate, %	36.0		35.7	
Poor to Undifferentiated, %	12.2		16.5	
Not specified, %	26.2		17.0	
Number of nodules		0		0
1, %	51		47.9	
2–3, %	23.1		19.6	
4+, %	7.5		14.9	
Not determined, %	18.4		17.6	
Size of largest nodule, cm	7.5(4.3)	19.4	7.9(4.5)	17.9
Nodule > 5 cm, %	77.6	0	77.9	0
Satellite lesions, %	17.0	2.4	19.1	3.2
Extrahepatic disease, %	24.5	2.7	24.3	4.2
Metastasis		0		0
Yes, %	19.4		19.9	
Not determined, %	78.2		75.9	
BCLC		11.2		12.2
Early (0 or A)	26.2		22.6	
Intermediate (B)	24.8		20.8	
Advanced (C or D)	37.8		44.4	
AFP at diagnosis, ng/mL				
<20, %	29.3		29.0	
20–399, %	33.3		34.0	
400+, %	37.4		37.0	
Tumor type		41.8		44.2
Classic, %	26.3		27.6	
Fibrolamellar, %	4.7		1.8	
Clear cell, %	1.8		3.1	
Other, %	4.7		3.4	
Not determined, %	62.6		64.0	

* Values are mean (SD) for continuous variables and percentage (95% confidence interval) for categorical variables. The values of the categorical variables may not add up to 100% because of rounding. Abbreviations: Barcelona Clinic Liver Cancer (BCLC), alpha-fetoprotein (AFP).

## Data Availability

Data described in the manuscript, codebook, and analytic code will be made available upon reasonable request, pending applications, and approval.
